# Transient effect of single or repeated acute deoxynivalenol and zearalenone dietary challenge on fecal microbiota composition in female finishing pigs

**DOI:** 10.1017/S1751731120001299

**Published:** 2020-11

**Authors:** M. Le Sciellour, O. Zemb, A.-M. Serviento, D. Renaudeau

**Affiliations:** 1 PEGASE, INRAE, Agrocampus Ouest, 16 Le Clos Domaine de la Prise, Saint-Gilles 35590, France; 2 GenPhySE, Université de Toulouse, INRA, INPT, INP-ENVT, 24, chemin de Borde-Rouge – Auzeville Tolosane, Castanet Tolosan 31320, France

**Keywords:** swine, microbiome, mycotoxin, performance, acute challenge

## Abstract

Mycotoxins are a major contaminant of pig feed and have negative effects on health and performance. The present study investigated the impact of single or repeated acute challenges with a diet naturally contaminated with deoxynivalenol (**DON**) and zearalenone (**ZEN**) on growth performances of finishing pigs and their fecal microbiota composition. A total of 160 pigs (castrated males and females) in two successive batches were randomly divided into four experimental groups of 40 pigs each. The control group received a control finisher diet from 99 to 154 days of age. Challenged groups were subjected to a 7-day acute challenge by being fed a DON- and ZEN-contaminated diet (3.02 mg DON/kg feed and 0.76 mg ZEN/kg feed) at 113 days (group **DC**), 134 days (group **CD**) or both 113 and 134 days (group **DD**). Microbiota composition was analyzed via 16S rRNA sequencing from fecal samples collected from the 80 females at 99, 119, 140 and 154 days. Challenged pigs (i.e. groups DC, CD and DD) reduced their average daily feed intake by 25% and 27% (*P* < 0.001) and feed efficiency by 34% and 28% (*P* < 0.05) during the first and second mycotoxin exposure, respectively. Microbiota composition was affected by mycotoxin exposure (*P* = 0.07 during the first exposure and *P* = 0.01 during the second exposure). At the family level, mycotoxin exposure significantly (*P* < 0.05) decreased the relative abundances of *Ruminococcaceae*, *Streptococcaceae* and *Veillonellaceae* and increased that of *Erysipelotrichaceae* at both 119 and 140 days of age. After the 7-day DON/ZEN challenge, the relative abundance of 6 to 148 operational taxonomic units (**OTUs**) differed among the treatment groups. However, none of these OTUs changed in all treatment groups. Using 27 functional pathways, pigs exposed to DON/ZEN challenges could be distinguished from control pigs using sparse partial least squares discriminant analysis, with a 15% misclassification rate. Regarding the functionality of these predictors, two pathways were involved in detoxifying mycotoxins: drug metabolism and xenobiotic metabolism by cytochrome P450. In challenged pigs, microbiota composition returned to the initial state within 3 weeks after the end of a single or repeated DON/ZEN challenge, highlighting the resilience of the gut microbiome. The feeding and growth performances of the pigs during challenge periods were significantly correlated with biological pathways related to health problems and modifications in host metabolism. To conclude, short-term DON/ZEN challenges resulted in transient modifications in the composition and functions of fecal microbiota.

## Implications

The present study aimed to investigate short- and long-term effects of a mycotoxin challenge (based on deoxynivalenol and zearalenone) on microbiota composition in pigs and to look at the relationships between these changes and host performance during and after these challenges. The study also demonstrated the transient impact of an acute challenge on fecal microbiota composition, suggesting that pigs in a commercial context may recover quickly from this type of disturbance.

## Introduction

Deoxynivalenol (**DON**) and zearalenone (**ZEN**) are secondary metabolites produced by *Fusarium* spp. of fungi. These mycotoxins are known in livestock production since they impair animal health and growth when ingested via contaminated cereals, such as wheat and maize, in feed (Bouhet and Oswald, 2005; Antonissen *et al.*, 2014). In particular, exposure of pigs to DON injures the epithelium, which compromises their immune functions, results in an inflammatory response and may cause diarrhea (Bouhet and Oswald, 2005; Sobrova *et al.*, 2010). This results in reduced feed intake and growth rate (Andretta *et al.*, 2012; Serviento *et al.*, 2018). Modifications in pig microbiota composition have been observed after chronic ingestion of the mycotoxins fumonisin (Mateos *et al.*, 2018) and DON (Waché *et al.*, 2008). In the latter study, chronic exposure to DON resulted in transient modifications in the abundance of fecal aerobic mesophilic bacteria during the first week of the DON challenge (Waché *et al.*, 2008). However, short- and long-term impacts of single or repeated acute DON/ZEN challenges on pig fecal microbiota composition have not yet been described. *In vitro* studies demonstrated that the microbiota could detoxify DON (He, 1992; Kollarczik *et al.*, 1994; Eriksen *et al.*, 2002 and 2003; Young *et al.*, 2007). Thus, microbiota could help hosts to continue functioning despite mycotoxin exposure via feed. Consequently, we assume that animals’ ability to cope with a mycotoxin challenge may be related to microbiota composition and modifications of it. The present study first evaluated the impact of single or repeated acute DON/ZEN challenges on short- and long-term changes in fecal microbiota. It then explored the relationship between microbiota functions and variations in pig performance due to the DON/ZEN challenge.

## Materials and methods

### Animals and experimental design

The experiment was conducted on Pietrain × (Large White × Landrace) pigs 99 to 154 days old. The 160 pigs (80 castrated males and 80 females) were raised in two batches from April to October 2017 in experimental facilities at INRAE’s Unité Expérimentale Porcs de Rennes, located in Saint Gilles, France. Within each batch, pigs were randomly allotted to four experimental groups in a randomized complete block design. Within each batch, all the pigs were housed in one single pen. Each litter and sex was equally represented in each treatment. The experimental room was equipped with a weighing machine and the type of automatic precision feeders described by Pomar *et al.* (2011). Serviento *et al.* (2018) describe the experimental room in detail. During the challenge periods, the living areas of control and challenged pigs were separated to avoid cross-contamination via feces. Pigs had *ad libitum* access to water and feed.

**Figure 1:**
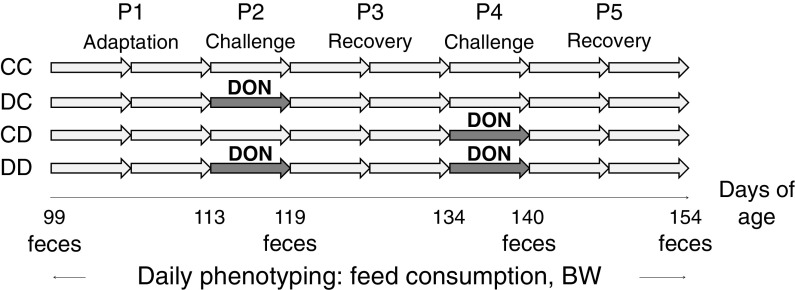
Experimental design. The pigs were given a control diet all over the experimental period (group CC) except for DC, CD and DD groups fed a deoxynivalenol- and zearalenone-contaminated diet (DON) during P2 (113 to 119 days), P4 (134 to 140 days) and both P2 and P4, respectively.

A naturally DON- and ZEN-contaminated feed was used to challenge the pigs. Both the control finisher diet and contaminated diet were based on maize and soybean meal, with 0.8 g standardized ileal digestible lysine per MJ of net energy, and met the ideal profile for essential amino acids. Serviento *et al.* (2018) describe the diet compositions in detail (online Supplementary Table S1). The control diet was based on maize, with trace DON concentration. The contaminated diet was formulated with naturally contaminated maize containing 4.8 mg DON/kg. According to analyses of mycotoxin composition, the control diet contained 0.14 mg DON/kg and 0.10 mg ZEN/kg, while the contaminated diet contained 3.02 mg DON/kg and 0.76 mg ZEN/kg. In the experimental design (Figure 1), the control group (**CC**) received the control finisher feed throughout the experiment (from 99 to 154 days of age). The challenged groups were subjected to the 7-day DON/ZEN challenge on day 113 (group **DC**), day 134 (group **CD**) or days 113 and 134 (group **DD**). Before and after the challenge periods, challenged pigs received the control finisher feed.

### Measurements

Live BW was recorded each time a pig passed through the weighing machine and was averaged daily into an average daily BW. The automatic feeders were used to record individual daily feed intake. Feces were collected from females at 99, 119, 140 and 154 days of age. Each fecal sample was taken directly from the rectum, immediately stored in barcode tubes and snap-frozen in liquid N_2_ within 10 min of sampling. All samples were stored at −80°C until DNA extraction.

### DNA sequencing

DNA was extracted from 50 mg samples of feces. Bead-beating lysis and DNA purification using the ZR-96 Soil Microbe DNA kit (Zymo Research, Irvine, CA, USA) were conducted according to the manufacturer instructions (Frese *et al.*, 2015). The V3-V4 region of the 16S rRNA gene was amplified through PCR using F460 and R460 primers (F460: CTTTCCCTACACGACGCTCTTCCGATCTACGGRAGGCAGCAG, R460: GGAGTTCAGACGTGTGCTCTTCCGATCTTACCAGGGTATCTAATCCT) and 30 cycles of PCR with an annealing temperature of 65°C. After sequencing on the Illumina MiSeq platform, the 2 × 250 bp paired-end sequences were cleaned internally for length, homopolymers and undetermined nucleotides. Sequencing and sequence cleaning were performed in the Get-PlaGE platform (Toulouse, France).

Chimeras were removed from the sequences using VSEARCH (Rognes *et al.*, 2016). The resulting sequences were clustered *de novo* into operational taxonomic units (**OTUs**) using a similarity threshold of 0.97, and the abundance of each OTU in each sample was recorded. Each OTU was assigned to a taxon based on the SILVA database using USEARCH (9.2.64_i86linux32 version). The PICRUSt pipeline was used to categorize KEGG (Kyoto Encyclopedia of Genes and Genomes) orthologs resulting from the 16S rRNA sequencing data into biological pathways.

### Calculations and statistical analysis

Statistical analyses were conducted using R 3.5.1. (R Core Team, 2018). Average daily gain (**ADG**), average daily feed intake (**ADFI**) and feed efficiency (**FE** = ADG/ADFI) of the female pigs were compared using ANOVA models with the following effects: period, treatment and their interaction. Each ANOVA model also included the fixed effect of the batch and the initial BW as a covariable.

Microbiota diversity was assessed based on the number of OTU and Shannon index using the *phyloseq* package of R (McMurdie and Holmes, 2013). The structure of the bacterial community was analyzed by calculating a Bray–Curtis distance matrix. The homogeneity of the dispersion within each group was checked using a multivariate analogue of Levene’s test for homogeneity of variances. A pairwise multivariate ANOVA (**MANOVA**) was applied to the distance matrix to compare the control and challenged groups at 99, 119, 140 and 154 days of age. Differences in groups’ bacterial communities were considered significant at an adjusted false discovery rate (***P***_**FDR**_) < 0.05 after 999 permutations based on the Benjamini–Hochberg procedure. In a second step, the impacts of the DON/ZEN challenges on microbiota composition at the family and genus levels were assessed using the non-parametric Wilcoxon test by comparing the relative abundance of family and genus of control and challenged groups at the end of each period (i.e. 99, 119, 140 and 154 days of age). Finally, the relative abundance of OTU of control and challenged pigs was compared using generalized linear models (**GLMs**) developed with the *edgeR* package of R (Robinson *et al.*, 2010). The GLMs included a batch effect within each treatment. The GLM analyses were conducted as pairwise comparisons between the control group and each treatment group within each period: at 99 days to assess differences before the challenges, at 119 and 140 days to assess short-term effects of DON/ZEN mycotoxins on microbiota composition and at 154 days to assess long-term effects of DON/ZEN mycotoxins. In the GLM analyses, abundances of an OTU were considered to differ significantly at *P*_FDR_ < 0.05.

To extract the biological functions that best distinguished control pigs from challenged pigs at 119 and/or 140 days of age, multivariate integrative sparse partial least squares discriminant analysis (**MINT sPLS-DA**) was used (Lê Cao *et al.*, 2011). This approach can aggregate functions even when they are provided by different microbial species. Using microbiota information collected at 119 and 140 days as two different datasets, this analysis was applied to the pathway abundance tables, which had been normalized using total-sum scaling and centered log-ratio transformation. The error rates of the resulting model were validated from 10 replicates of 10-fold cross-validation.

The ADG, ADFI and FE were averaged during the DON/ZEN challenges (113 to 119 and 134 to 140 days of age) and corrected for the batch effect and initial BW at 99 days of age using a linear model. The relationship between each pair of performance traits (ADG, ADFI and FE) was analyzed via Pearson correlations, and the correlations were considered significant at *P*_FDR_ < 0.05. Regularized canonical correlation analysis (**rCCA**) was used to analyze correlations between animal performance (ADG, ADFI and FE) and the relative abundance of biological pathways of the microbiota. As a multivariate approach, rCCA calculates correlations between two datasets acquired from the same experimental units when the datasets have more variables than samples (González *et al.*, 2008). To determine a significant threshold for the correlation of each performance–pathway pair, the matrix of the relative abundance of the pathways was randomized by the pathway. The correlation of each performance–pathway pair was then calculated and recorded. This sequence of randomization and correlation was repeated 100 times. For each pair, the threshold of a significant positive or negative correlation was the maximum or minimum correlation, respectively, obtained during 100 randomizations.

## Results

### Animal performance

Impacts of the DON/ZEN challenges on the performances of all pigs (males and females combined) were previously published (Serviento *et al.*, 2018). Thus, we provide results only for the performance of females because microbiota data were collected only from them. Mean (±1 SD) BW was 54.2 ± 5.0 kg at 99 days of age and 106.3 ± 10.0 kg at 154 days. Briefly, when exposed to a DON/ZEN challenge, mean ADFI and ADG decreased significantly. For instance, ADFI decreased from 2.39 to 1.74 kg/day from days 113 to 119, respectively, and 2.91 to 2.17 kg/day from days 134 to 140, respectively (*P* < 0.001) (itemized values are presented in Table 1). However, mean ADFI and ADG did not differ between the control and challenged pigs over the entire period (ADG: 0.98 and 0.94 kg/day, respectively; *P* > 0.05). Mean FE decreased during DON/ZEN challenge periods (from 0.44 to 0.29 from days 113 to 119, respectively, and from 0.40 to 0.29 from days 134 to 140, respectively; *P* < 0.05). However, mean FE did not differ between control and challenged pigs during recovery periods (i.e. 0.39 *v*. 0.41, respectively, from 119 to 134 days of age; 0.34 and 0.37, respectively, from 140 to 154 days of age; *P* > 0.05).

**Table 1:** Effect of the mycotoxin challenge on the pigs performance

	Treatments^1^				RSD^2^	Statistics
	CC	DC	CD	DD		
No. of pigs	15	16	15	18		
ADFI (kg/day)						
P1 (99 to 112 days)	2.19^u^	2.26^uv^	2.24^u^	2.13^u^	0.37	P***, PxT***
P2 (113 to 119 days)	2.43^au^	1.83^bu^	2.35^au^	1.64^bv^		
P3 (120 to 133 days)	2.58^uv^	2.72^vw^	2.56^u^	2.55^w^		
P4 (134 to 140 days)	2.92^av^	3.10^aw^	2.28^bu^	2.07^buv^		
P5 (141 to 154 days)	3.01^v^	3.10^w^	3.13^v^	3.18^x^		
P1 to P5	2.62	2.65	2.57	2.43	0.30	ns
ADG (kg/day)						
P1	0.77^u^	0.85^v^	0.78^u^	0.76^u^	0.21	P***, PxT***, PxB**
P2	1.10^av^	0.52^bu^	1.01^avw^	0.49^bv^		
P3	0.97^u^	1.11^vw^	1.02^vw^	1.08^w^		
P4	1.16^av^	1.19^aw^	0.65^bu^	0.65^buv^		
P5	1.01^u^	1.07^vw^	1.15^w^	1.15^w^		
P1 to P5	0.98	0.97	0.95	0.89	0.12	ns
FE						
P1	0.36^u^	0.38^u^	0.35^uv^	0.36^uv^	0.08	PxT***, PxB**
P2	0.46^av^	0.27^bv^	0.42^au^	0.30^bu^		
P3	0.38^uv^	0.41^u^	0.40^u^	0.42^v^		
P4	0.40^auv^	0.39^au^	0.28^bv^	0.31^abu^		
P5	0.33^u^	0.34^uv^	0.37^uv^	0.36^uv^		
P1 to P5	0.37	0.37	0.37	0.37	0.03	ns

ADFI = average daily feed intake; ADG = average daily gain; FE = feed efficiency.

^a,b^Least square means within a row with different superscript differ according to the treatment group.

^u,v,w,x^Least square means within a column with different superscript differ according to the period.

1 Control pigs (CC) were fed a control diet. Pigs in the challenged groups were fed a deoxynivalenol- and zearalenone-contaminated diet between 113 and 119 days (DC), between 134 and 140 days (CD) and both between 113 and 119 days and between 134 and 140 days (DD).

2 Residual SD from an ANOVA model accounting for the period (P), the treatment (T), the batch (B) and their interactions. The initial BW was included as a covariable in the models. **P < 0.01, ***P < 0.001, ns = not significant.

### Microbiota composition

A total of 277 fecal samples were collected from 74 female pigs. Six females were removed from the experiment due to health problems or their inability to adapt to the sorting machine or automatic feeders. In addition, we were not able to collect fecal samples from some female pigs. The number of sequences in the samples ranged from 15 924 to 76 582 (mean ± 1 SD = 29 482 ± 11 282). No sample was discarded due to having too few sequences. After filtering out rare OTUs (<0.01% of all sequences in the dataset), 1556 OTUs remained in the dataset.

At 99 days of age, mean ± 1 SD of OTU abundance of the fecal samples was 3649 ± 3642, while the mean ± 1 SD Shannon diversity index was 7.13 ± 0.16 (Table 2). Based on the Wilcoxon tests applied to the richness and the Shannon index of the control and challenged pigs at 119 or 140 days of age, the mycotoxin exposure did not impact microbial diversity (*P* > 0.05) (Table 2). *Firmicutes* was the most abundant phylum (80.10% of the affiliated sequences), followed by *Bacteroidetes* (15.37%). *Actinobacteria*, *Proteobacteria* and *Spirochaetes* were represented by 0.69%, 0.49% and 0.18% of the sequences, respectively. *Tenericutes* and *Fibrobacteres* each represented less than 0.01% of the sequences. The remaining sequences were unclassified.

**Table 2:** Diversity indexes (mean ± SD) and relative abundance (mean ± SD) of families and genera in pigs’ fecal samples collected in the four different experimental groups^1^ at 99, 119, 140 and 154 days of age

	d99	d119			d140			d154
	All^2^	Ctrl^3^	DON/ZEN^3^	*P* ^4^	Ctrl^5^	DON/ZEN^5^	*P* ^6^	All^2^
Number of pigs	71	31	35		34	37		72
Diversity indexes								
Richness (number of operational taxonomic units)	3649 ± 364	3653 ± 279	3507 ± 385	0.10	3764 ± 261	3626 ± 347	0.10	3746 ± 295
Shannon index	7.13 ± 0.16	7.14 ± 0.14	7.13 ± 0.16	0.91	7.21 ± 0.07	7.16 ± 0.13	0.05	7.18 ± 0.11
Relative abundance (%)								
Families								
*Desulfovibrionaceae*	0.05 ± 0.06	0.05 ± 0.04	0.08 ± 0.06	0.11	0.07 ± 0.04	0.10 ± 0.06	<0.01	0.08 ± 0.06
*Erysipelotrichaceae*	0.57 ± 0.40	0.98 ± 0.54	1.61 ± 0.81	<0.001	1.23 ± 0.67	1.90 ± 1.22	<0.05	1.45 ± 0.94
*Lachnospiraceae*	9.28 ± 2.60	5.70 ± 2.03	5.35 ± 2.60	0.36	5.45 ± 1.77	4.35 ± 1.97	<0.05	4.24 ± 2.14
*Peptostreptococcaceae*	0.04 ± 0.03	0.14 ± 0.06	0.19 ± 0.09	<0.05	0.55 ± 0.21	0.68 ± 0.35	0.18	0.62 ± 0.35
*Prevotellaceae*	7.41 ± 4.26	5.97 ± 4.07	5.02 ± 4.31	0.21	5.49 ± 4.66	2.74 ± 2.59	<0.01	2.61 ± 2.59
*Ruminococcaceae*	4.42 ± 1.54	2.78 ± 0.91	2.36 ± 1.45	<0.05	2.53 ± 1.16	1.76 ± 0.84	<0.01	1.80 ± 0.96
*Streptococcaceae*	13.76 ± 6.07	9.86 ± 5.17	5.29 ± 4.19	<0.001	9.60 ± 3.78	5.49 ± 3.67	<0.001	9.21 ± 4.62
*Veillonellaceae*	1.79 ± 1.75	0.88 ± 1.19	0.27 ± 0.37	<0.001	0.36 ± 0.57	0.13 ± 0.19	<0.01	0.12 ± 0.18
Genera								
*Bacteroides*	0.00 ± 0.00	0.00 ± 0.00	0.01 ± 0.05	0.96	0.00 ± 0.01	0.00 ± 0.00	<0.05	0.05 ± 0.20
*Blautia*	1.96 ± 0.81	0.80 ± 0.32	0.70 ± 0.55	<0.05	0.54 ± 0.25	0.62 ± 0.41	0.74	0.51 ± 0.33
*Clostridium IV*	0.02 ± 0.02	0.04 ± 0.03	0.06 ± 0.05	<0.01	0.06 ± 0.04	0.06 ± 0.03	0.61	0.07 ± 0.04
*Clostridium XI*	0.02 ± 0.02	0.09 ± 0.05	0.14 ± 0.08	<0.01	0.57 ± 0.31	0.55 ± 0.25	0.92	0.57 ± 0.32
*Dialister*	1.32 ± 1.21	0.63 ± 0.89	0.12 ± 0.26	<0.001	0.14 ± 0.36	0.16 ± 0.33	0.73	0.08 ± 0.16
*Fusicatenibacter*	0.37 ± 0.20	0.21 ± 0.13	0.14 ± 0.14	<0.05	0.12 ± 0.12	0.15 ± 0.12	0.20	0.11 ± 0.11
*Megasphera*	0.02 ± 0.02	0.00 ± 0.01	0.00 ± 0.00	<0.05	0.00 ± 0.00	0.00 ± 0.00	0.59	0.00 ± 0.01
*Mitsuokella*	0.35 ± 0.48	0.15 ± 0.27	0.03 ± 0.07	<0.001	0.03 ± 0.08	0.02 ± 0.05	0.32	0.01 ± 0.02
*Olsenella*	0.47 ± 1.02	0.12 ± 0.24	0.03 ± 0.06	<0.01	0.01 ± 0.02	0.00 ± 0.00	0.07	0.00 ± 0.01
*Ruminococcus*	0.23 ± 0.14	0.36 ± 0.24	0.23 ± 0.13	<0.05	0.37 ± 0.24	0.34 ± 0.25	0.59	0.31 ± 0.28
*Sarcina*	0.19 ± 0.27	0.21 ± 0.20	0.56 ± 0.64	<0.05	0.23 ± 0.35	0.29 ± 0.35	0.38	0.14 ± 0.26
*Streptococcus*	13.76 ± 6.07	9.86 ± 5.17	5.29 ± 4.19	<0.001	7.47 ± 4.37	7.27 ± 4.14	0.90	9.21 ± 4.62
*Turicibacter*	0.45 ± 0.40	0.90 ± 0.56	1.55 ± 0.83	<0.001	1.61 ± 1.24	1.49 ± 0.90	0.87	1.41 ± 0.96

1 The treatment groups are: the CC group fed a control diet during the whole experiment, the DC group fed a deoxynivalenol (DON)- and zearalenone (ZEN)-contaminated diet between 113 and 119 days, the CD group fed a DON- and ZEN-contaminated diet between 134 and 140 days, and the DD group fed a DON- and ZEN-contaminated diet between 113 and 119 days and between 134 and 140 days.

2 All pigs were in control conditions at 99 and 154 days of age.

3 At 119 days of age, Ctrl referred to the pigs in the CC and CD groups, and DON referred to the pigs in the DC and DD groups.

4 The *P*-value resulted from a Wilcoxon test between Ctrl and challenged pigs at 119 days of age.

5 At 140 days of age, Ctrl referred to the pigs in the CC and DC groups, and DON/ZEN referred to the pigs in the CD and DD groups.

6 The *P*-value resulted from a Wilcoxon test between Ctrl and challenged pigs at 140 days of age.

According to the Wilcoxon tests, eight families were impacted by the DON/ZEN challenges (Table 2). Compared to the control pigs, mycotoxin exposure from days 113 to 119 decreased the relative abundances of *Ruminococcaceae* (*P* < 0.05), *Streptococcaceae* (*P* < 0.001) and *Veillonellaceae* (*P* < 0.001), and increased those of *Erysipelotrichaceae* (*P* < 0.001) and *Peptostreptococcaceae* (*P* < 0.05). Mycotoxin exposure from days 134 to 140 decreased the relative abundances of *Lachnospiraceae* (*P* < 0.05), *Prevotellaceae* (*P* < 0.01), *Ruminococcaceae* (*P* < 0.01), *Streptococcaceae* (*P* < 0.001) and *Veillonellaceae* (*P* < 0.01), and increased those of *Desulfovibrionaceae* (*P* < 0.01) and *Erysipelotrichaceae* (*P* < 0.05). At the genus level, mycotoxin exposure from days 113 to 119 decreased the relative abundances of *Blautia* (*P* < 0.05), *Dialister* (*P* < 0.001), *Fusicatenibacter* (*P* < 0.05), *Mitsuokella* (*P* < 0.001), *Olsenella* (*P* < 0.01), *Ruminococcus* (*P* < 0.05) and *Streptococcus* (*P* < 0.001), and increased those of *Clostridium IV* (*P* < 0.01), *Clostridium XI* (*P* < 0.01), *Sarcina* (*P* < 0.05) and *Turicibacter* (*P* < 0.001). Mycotoxin exposure from days 134 to 140 did not significantly influence the relative abundance of genera. However, the relative abundance of two genera (*Lactobacillus* and *Collinsella*) and three families (*Lactobacillaceae*, *Coriobacteriaceae* and *Spirochaetaceae*) differed significantly between groups CD and DD at 140 days (online Supplementary Table S2).

**Figure 2:**
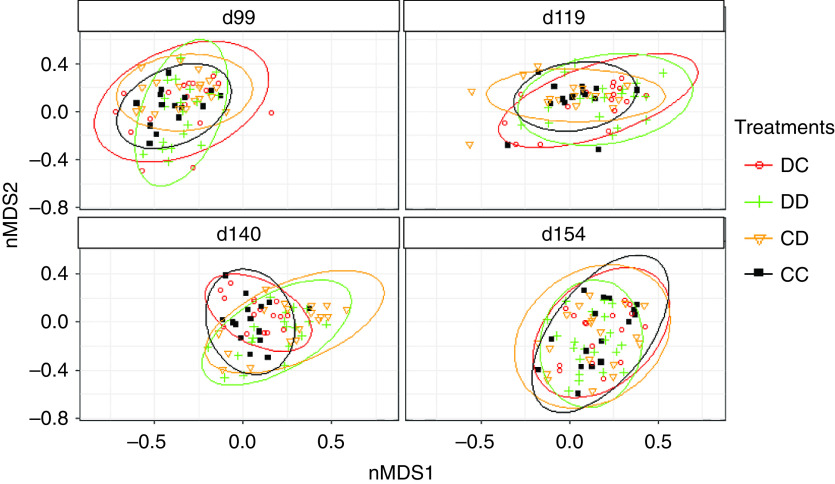
Bray–Curtis distance between treatment groups and controls at 99, 119, 140 and 154 days of age represented in a non-metric multidimensional scaling (nMDS). The control group (CC) was compared with treatment groups: group of pigs fed a deoxynivalenol (DON)- and zearalenone (ZEN)-contaminated diet between 113 and 119 days (DC), group fed a DON- and ZEN-contaminated diet between 134 and 140 days (CD), group fed a DON- and ZEN-contaminated diet between 113 and 119 days and between 134 and 140 days (DD). The *P*-values are presented in Table 1.

According to the MANOVA, microbiota composition did not differ among control or treatment groups (*P*_FDR_ > 0.52) at the beginning of the experiment (99 days of age) (Figure 2, Table 3). At the end of the first challenge period, microbiota composition of challenged pigs tended to differ from that of control pigs (*P* = 0.07). At the end of the second challenge period, microbiota composition of challenged pigs differed significantly from that of control pigs (*P* = 0.01). After 7 days of DON/ZEN challenge, microbiota composition did not differ between groups DC and DD at 119 days (*P*_FDR_ = 0.61) or between groups CD and DD at 140 days (*P*_FDR_ = 0.43). After a 2-week recovery period, at 154 days, microbiota composition did not differ among control or treatment groups (*P*_FDR_ = 0.99).

**Table 3:** Adjusted false discovery rate *P*-values (*P*_FDR_) using Benjamini–Hochberg method resulting from 999 iterations of a pairwise multivariate ANOVA between control (CC) and treatment groups of pigs exposed to a deoxynivalenol- and zearalenone-contaminated diet

Days of age	Number of pigs	*P*_FDR_				
		DC *v.* CC	CD *v.* CC	DD *v.* CC	DC *v*. DD	CD *v*. DD
d99	71	0.52	0.52	0.68	0.72	0.58
d119	66	0.07	0.82	0.07	0.61	0.06
d140	68	0.49	0.01	0.01	0.01	0.43
d154	72	0.99	0.99	0.99	0.99	0.99

Pigs in the treatment groups were fed a deoxynivalenol- and zearalenone-contaminated diet between 113 and 119 days (DC), between 134 and 140 days (CD) and both between 113 and 119 days and between 134 and 140 days (DD).

**Figure 3:**
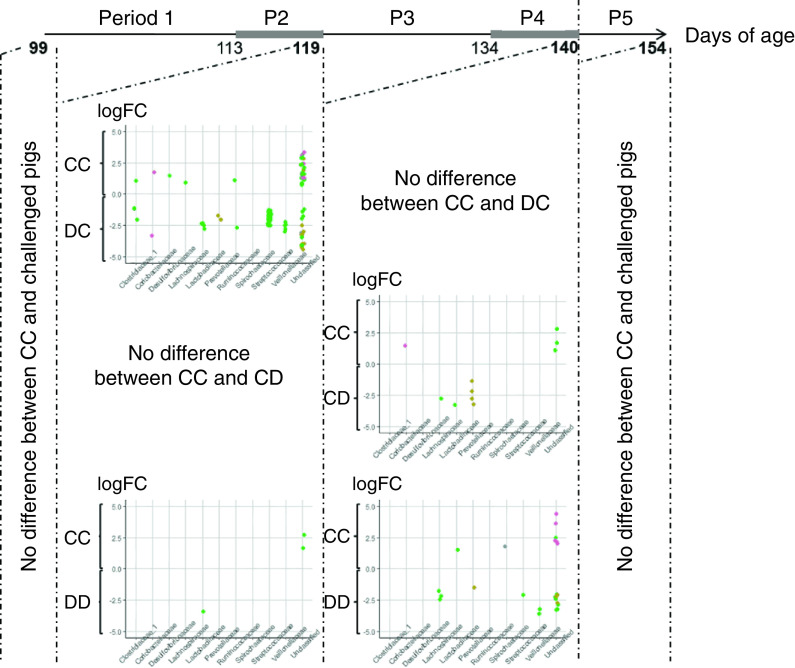
Operational taxonomic units differentially abundant between control group (CC) and treatment groups: group of pigs fed a deoxynivalenol (DON)- and zearalenone (ZEN)-contaminated diet between 113 and 119 days (DC), group fed a DON- and ZEN-contaminated diet between 134 and 140 days (CD), group fed a DON- and ZEN-contaminated diet between 113 and 119 days and between 134 and 140 days (DD). Log2 fold changes (logFC) resulted from a 2 × 2 comparison using a generalized linear model analysis (*P* < 0.05).

According to the MANOVA, the global microbiota composition recovers within 2 weeks after the end of a DON/ZEN challenge. Then, we considered that the GLM analyses could be applied at each age without introducing unwanted biases. In the GLM analysis, relative OTU abundance did not differ between control and challenged pigs at 99 days (Figure 3). At 119 days, relative abundance of 148 of the 1556 OTUs in the dataset differed between groups CC and DC and 6 between groups CC and DD. At 140 days, relative abundance of 15 OTUs differed between groups CC and CD, 32 between groups CC and DD and 2 between groups CD and DD. None of the OTUs that differed in relative abundance after the first challenge period (day 119) also differed after the second challenge period (day 140), and viceversa. After the recovery period, at 154 days, relative OTU abundance did not differ between control and challenged pigs.

**Figure 4:**
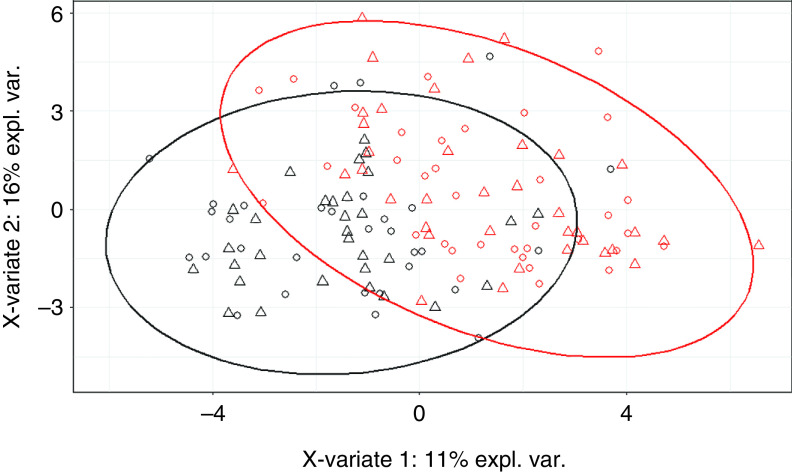
Multivariate integrative sparse partial least squares discriminant analysis based on pathways abundance at 119 days (○) and 140 days (Δ). Pigs receiving a deoxynivalenol (DON)- and zearalenone (ZEN)-contaminated diet (in red) were discriminated from the control pigs (in black) with 27 pathways and 15% error rate of misclassification. Expl. var. refers to *t* amount of variation explained per component.

Based on pathway abundance, the MINT sPLS-DA distinguished the pigs that received the contaminated diet from control pigs using 27 pathways, with a 15% misclassification rate (Figure 4). Based on loading values, the main pathways that contributed to the first component of the sPLS-DA were lipid metabolism, amyotrophic lateral sclerosis, amoebiasis, RIG-I-like receptor signaling, and carbohydrate digestion and absorption (online Supplementary Table S3). The main pathways that contributed to the second component were retinol metabolism, drug metabolism by cytochrome P450, xenobiotic metabolism by cytochrome P450, biosynthesis of siderophore group non-ribosomal peptides and inositol phosphate metabolism (online Supplementary Table S3).

### Relationships between performance and microbial functioning

For correlations among performance traits, ADG correlated positively with ADFI from days 113 to 119 (P2) for control pigs (*r* = 0.63, *P* < 0.001) and challenged pigs (*r* = 0.49, *P* < 0.05), and from days 134 to 140 (P4) for control pigs (*r* = 0.46, *P* < 0.01) and challenged pigs (*r* = 0.68, *P* < 0.001).

Based on the rCCA, correlation coefficients between performance traits of control or challenged pigs during P2 and P4 and the relative abundance of microbiota pathways depended on the trait and the period. Microbiota pathways were weakly correlated with ADG or FE of the control and challenged pigs (Table 4). Compared to P2, fewer correlations were significant during P4. During P2, ADFI was significantly correlated with 11 pathways in the challenged pigs, but with no pathways in the control pigs (Table 4). The 11 pathways corresponded to functions were related to environmental information processing, the organismal system, human diseases and metabolism (terpenoid and polyketide metabolism, carbohydrate and glycan metabolism, and xenobiotic biodegradation and metabolism). No significant correlation with ADFI was found during P4 during the second challenge. ADG and FE were not correlated with the relative abundance of pathways during the challenge periods. Feed efficiency of control pigs was correlated with two pathways during P2, but ADFI and ADG showed no significant correlations. However, for control pigs during P4, ADFI was correlated with three pathways and ADG with one pathway. No pathway was correlated with any performance indicator of either control or challenged pigs (Table 4).

**Table 4:** Regularized canonical correlations (rCCA)^1^ between functional pathways relative abundance and the average daily feed intake (ADFI)^2^, the average daily gain (ADG) and the feed efficiency (FE) of pigs

Function level A	Function level B	Pathway	ADFI				ADG				FE			
			Control^3^		Challenged^4^		Control		Challenged		Control		Challenged	
			P2^5^	P4^6^	P2	P4	P2	P4	P2	P4	P2	P4	P2	P4
Environmental information processing	Membrane transport	Secretion system	–	–	−0.52	–	–	–	–	–	–	–	–	–
Organismal system	Circulatory system	Cardiac muscle contraction	–	–	0.43	–	–	–	–	–	–	–	–	–
Human diseases	Cancers	Bladder cancer	–	–	−0.47	–	–	–	–	–	–	–	–	–
	Immune diseases	Primary immunodeficiency	–	–	–	–	–	–	–	–	0.47	–	–	–
	Neurodegenerative diseases	Huntington’s disease	–	–	−0.44	–	–	–	–	–	–	–	–	–
Metabolism	Carbohydrate metabolism	Butanoate metabolism	–	–	−0.48	–	–	–	–	–	–	–	–	–
	Glycan biosynthesis and metabolism	Other glycan degradation	–	–	0.46	–	–	–	–	–	–	–	–	–
		Glycosaminoglycan degradation	–	–	–	–	–	0.46	–	–	–	–	–	–
	Lipid metabolism	Linoleic acid metabolism	–	−0.44	–	–	–	–	–	–	–	–	–	–
		Primary bile acid biosynthesis	–	–	–	–	–	–	–	–	0.55	–	–	–
	Metabolism of other amino acids	Phosphonate and phosphinate metabolism	–	–	−0.43	–	–	–	–	–	–	–	–	–
	Metabolism of terpenoids and polyketides	Limonene and pinene degradation	–	–	−0.49	–	–	–	–	–	–	–	–	–
		Benzoate degradation	–	–	−0.48	–	–	–	–	–	–	–	–	–
		Tetracycline degradation	–	–	−0.41	–	–	–	–	–	–	–	–	–
	Xenobiotics biodegradation and metabolism	Ethylbenzene degradation	–	–	−0.47	–	–	–	–	–	–	–	–	–
		Bisphenol degradation	–	−0.43	–	–	–	–	–	–	–	–	–	–
		Toluene degradation	–	0.43	–	–	–	–	–	–	–	–	–	–

1 To determine a significant threshold to each performance–pathway pair, the matrix of the relative abundance of the pathways was randomized by pathway. A rCCA was then applied, and each correlation by pair was kept. This randomization followed by rCCA was repeated 1000 times. For each pair, the thresholds for a significant positive or negative correlation were the maximum or minimum correlation obtained during the 1000 randomizations, respectively. This table presents all and only the significant correlations.

2 The performances (ADFI, ADG and FE) were averaged during the deoxynivalenol (DON)/zearalenone (ZEN) challenge periods (P2 and P4) and corrected for the batch effect and the initial BW at 99 days of age using a linear model. The square mean values of ADFI, ADG and FE were used for the rCCA.

3 Pathways significantly correlated with performance (ADFI, ADG and FE) in control pigs but not in challenged pigs.

4 Pathways significantly correlated with performance (ADFI, ADG and FE) in challenged pigs but not in control pigs.

5 The treatment groups are: the CC group fed a control diet during the whole experiment, the DC group fed a DON- and ZEN-contaminated diet between 113 and 119 days, the CD group fed a DON- and ZEN-contaminated diet between 134 and 140 days, and the DD group fed a DON- and ZEN-contaminated diet between 113 and 119 days and between 134 and 140 days. In P2 (between 113 and 119 days of age), the control pigs belong to the CC and CD experimental groups and the challenged pigs belonged to the DC and DD groups. The performances were correlated with the microbial data at 119 days of age.

6 In P4 (between 134 and 140 days of age), the control pigs belong to the CC and DC experimental groups and the challenged pigs belonged to the CD and DD groups. The performances were correlated with the microbial data at 140 days of age.

## Discussion

Because the impact of DON/ZEN challenges on pig performance was previously described (Serviento *et al.*, 2018), the present study focused mainly on the challenges’ impacts on gut microbiota and the latter’s relationship with pig performance. Regardless of age at exposure to DON and ZEN, the relative abundances of the families *Streptococcaceae*, *Ruminococcaceae* and *Veillonellaceae* decreased and that of *Erysipelotrichaceae* increased. These families were consistently impacted by the DON/ZEN challenge. In contrast, Reddy *et al.* (2018a) observed no difference in these families in 8-week-old castrated pigs subjected to a 4-week DON challenge. This difference from the present study could be related to the difference in sex (castrated males *v*. females), age of the pigs (8 *v*. 17 weeks old), breed or environmental factors such as diet or location. At the sub-family level, the OTUs or genera in the present study that were impacted by the DON/ZEN challenge at 119 days of age differed from those impacted at 140 days of age. This confirmed that the effects of acute DON/ZEN exposure on microbiota composition are time-dependent.

The present study was designed to evaluate the effect of repeated DON/ZEN challenges on microbiota composition. According to its results, the relative abundance of only two OTUs differed between groups CD and DD at 140 days of age. In addition, the relative abundance of only two genera (*Lactobacillus* and *Collinsella*) and their corresponding families differed significantly between groups CD and DD at 140 days of age. This result was confirmed by the MANOVA, which suggests that no habituation mechanisms were observed in microbiota composition when pigs were challenged twice. Accordingly, and based on the ADG and ADFI observed, pigs that had experienced a previous DON/ZEN challenge (group DD) performed similar to those challenged only once (group CD) (Serviento *et al.*, 2018). At a higher DON concentration (>5 mg/kg BW) (Flannery *et al.*, 2011), however, higher ADFI was observed in mice challenged twice. Despite differences in species, diet and other raising conditions, these studies could suggest a dose-dependent effect of DON on host responses and habituation.

In contrast to the effects of chronic ingestion of DON (Robert *et al.*, 2017), our results indicate that acute exposure to a DON- and ZEN-contaminated diet has no detrimental effects on overall pig ADG and FE after a 2-week recovery period. Based on patterns in ADFI, pigs given the DON- and ZEN-contaminated diet started to consume similar amounts than the pigs given the control diet after 3 days of challenge (Serviento *et al.*, 2018). This resulted in a full performance recovery at the end of the trial. With a bit less naturally contaminated diet (2.8 *v*. 3.02 mg DON/kg; 0.28 *v* 0.76 mg ZEN/kg), Waché *et al.* (2008) observed that pigs recovered full performance after 1 week exposure. Then, we assume that consuming a diet naturally contaminated with DON and ZEN at the concentrations under 3.02 and 0.76 mg/kg, respectively, during 1 week will result in a full performance recovery. Looking at the microbiota composition between control and challenged pigs 1 week after the beginning of the challenge, Waché *et al.* (2008) did not highlight any difference. Based on our results obtained with a bit more contaminated diet, the number of OTUs that differed in relative abundance between the control pigs and pigs after 1 week of exposure (119 or 140 days of age) ranged from 6 to 148 out of 1556 OTUs in the dataset. This clearly suggests that microbiota composition in the present study also changed moderately after a 7-day exposure to DON and ZEN.

Depending on the level of contamination, 45% to 95% of the DON ingested by pigs end up in the urine without being transformed by the microbiota (Prelusky *et al.*, 1988; Eriksen *et al.*, 2003; Dänicke *et al.*, 2004; Nagl *et al.*, 2014). In other words, gut microbiota have the opportunity to detoxify 5% to 55% of the DON ingested. Similarly, ca. 26% of ZEN end up in the urine (Binder *et al.*, 2017), which could explain in part the moderate changes in fecal microbiota composition observed in the present study during acute mycotoxin exposure. This agrees with previous studies in which fecal microbiota composition changed moderately during chronic DON exposure in pigs (Waché *et al.*, 2008), or not at all in rodents (Saint-Cyr *et al.*, 2013; Payros *et al.*, 2017).

As mentioned, relative bacterial abundance changed moderately by the end of the DON/ZEN challenges, but these differences were no longer present 2 weeks later. This clearly indicates that short-term exposure to DON and ZEN had a short and transient impact on microbiota composition in pigs. The lack of a long-term effect of DON/ZEN contamination could be explained in part by pigs’ rapid elimination of mycotoxins. Half of the ingested DON is excreted in the urine within 5.8 h (Dänicke *et al.*, 2004), and all is excreted within 24 h (Prelusky *et al.*, 1988).

The pathways extracted from the sPLS-DA differed from those that were significantly correlated with the performances. This lack of common pathways highlights limits of a functional interpretation of the variables extracted from the discriminant analysis. The information used to distinguish groups does not always have biological significance or utility and should be interpreted with caution. Nevertheless, on a short-term basis, changes in relative OTU abundance due to DON/ZEN exposure were due to a slight increase in biological functions related to drug and xenobiotic metabolism by cytochrome P450 (pathways used in the discriminant analysis). This function restores gut health by degrading toxic compounds such as xenobiotic mycotoxins (Sobrova *et al.*, 2010; Wilson and Nicholson, 2017). In the present study, some *Clostridium* bacteria were impacted by the DON challenge. In chickens, the *Clostridiales* can de-epoxify up to 100% of DON into a much less toxic form: DOM-1 (Yu *et al.*, 2010). This ability of microbiota to detoxify DON was previously observed in chickens (Yu *et al.*, 2010; Pierron *et al.*, 2016), humans (Gratz *et al.*, 2013) and pigs (Eriksen *et al.*, 2002; Gratz *et al.*, 2018). In an *in vitro* study, Chlebicz and Śliżewska (2019) identified *Lactobacillus* strains that can detoxify DON, ZEN and other mycotoxins. These results confirm that the microbiota seems to play a key role in detoxifying mycotoxins. In livestock species, growing evidence suggests a connection between microbiota composition and host performance. However, it is unclear whether a change in the functioning of gut microbiota is the cause or the consequence of variations in host performance.

As mentioned, microbiota could help hosts to maintain good health when exposed to mycotoxins, with subsequent positive effects on performance. Some microbial functions were significantly correlated with ADFI only during challenge periods. We hypothesize that mycotoxin challenge exacerbated differences between the pigs and emphasized significant correlations between performance and the microbial functions. It can be hypothesized that changes in animal performance, especially ADFI, due to mycotoxin exposure would also affect the composition of gut microbiota. In the present study, one microbiota functional pathway related to xenobiotic biodegradation and metabolism and three pathways related to the metabolism of terpenoids and polyketides (from which the mycotoxins originated (Huffman *et al.*, 2010)) were negatively correlated with ADFI during the first challenge period. This suggests that the pigs with ADFI decreased by the DON/ZEN challenge had increased abundance of these microbial functions. Specifically, some changes in the functioning of gut microbiota were likely related indirectly to the effect of DON/ZEN mycotoxins on ADFI, rather than related directly to the effect of the mycotoxins on microbiota composition.

Two pathways related to human diseases were significantly correlated with ADFI of challenged pigs. Although the meaning of these pathways in pigs is questionable, we assume that these modifications were related to disturbance in the health status of hosts that ingested DON/ZEN mycotoxins. Chronic ingestion of mycotoxins has impacted the health of human and pig hosts: intestinal pathologies, disturbance of intestinal barrier function and changes in the immune response (Pinton and Oswald, 2014; Robert *et al.*, 2017). Effects are particularly strong for pigs: 4-week chronic exposure of 8 mg DON/kg or 0.8 mg ZEN/kg in the feed changed the immune response and resulted in a decrease in ADFI and BW (Reddy *et al.*, 2018b). The additional energy cost of the inflammation response reduced the amount of energy available for growth.

## Conclusion

Single or repeated short-term DON/ZEN challenges resulted in transient modifications in the composition of fecal microbiota and microbial biological functions related to mycotoxin detoxification. The challenges had no effect on long-term changes in microbiota composition. Functions related to metabolism and health status were correlated with ADFI when pigs were exposed to a mycotoxin challenge, which suggests that the microbiota helped their host manage the mycotoxin disturbance.

## Software and data repository resources

None of the data were deposited in an official repository.

## Supplementary material

For supplementary material accompanying this paper visit https://doi.org/10.1017/S1751731120001299. click here to view supplementary material
