# Supplementary material for: Transient effect of single or repeated acute deoxynivalenol and zearalenone dietary challenge on fecal microbiota composition in female finishing pigs
Source: Animal. 2020 Jun 18;14(11):2277–87. doi: 10.1017/S1751731120001299 (PMC7538342; doi:10.1017/S1751731120001299)
Supplement: Supplementary file 1 [file S1751731120001299sup.zip › S1751731120001299sup001.docx]

**Transient effect of single or repeated acute deoxynivalenol and zearalenone dietary challenge on fecal microbiota composition in female finishing pigs**

M. Le Sciellour, O. Zemb, A.-M. Serviento and D. Renaudeau

*animal* journal

Table S3 Pathways contributing to the construction of the two first axis of the MINT sparse Partial Least Square Discriminant Analysis based on microbial pathways abundance at 119 days and 140 days. Pigs receiving a deoxynivalenol- and zearalenone-contaminated diet were discriminated from the control pigs with 27 pathways and 15% error-rate of misclassification.

| pathway | axis1 | axis2 |
| --- | --- | --- |
| Amoebiasis | 0.40157096 | 0.00000000 |
| Amyotrophic lateral sclerosis ALS | 0.42177637 | 0.00000000 |
| Ascorbate and aldarate metabolism | -0.24516364 | 0.00000000 |
| Bisphenol degradation | 0.02580230 | 0.00000000 |
| Carbohydrate digestion and absorption | -0.32780907 | 0.00000000 |
| Chloroalkane and chloroalkene degradation | 0.18507882 | 0.00000000 |
| Drug metabolism cytochrome P450 | 0.07584680 | 0.45862556 |
| Electron transfer carriers | 0.06688102 | 0.00000000 |
| Ether lipid metabolism | -0.42392811 | 0.00000000 |
| Flavone and flavonol biosynthesis | -0.24805558 | 0.00000000 |
| G protein coupled receptors | 0.09042758 | 0.00000000 |
| Galactose metabolism | -0.04015747 | 0.00000000 |
| Naphthalene degradation | 0.07064304 | 0.00000000 |
| Non homologous end joining | 0.21579645 | 0.00000000 |
| Penicillin and cephalosporin biosynthesis | -0.13038998 | 0.00000000 |
| Phosphotransferase system PTS | -0.08687196 | 0.00000000 |
| Prion diseases | 0.07241640 | 0.00000000 |
| RIG I like receptor signaling pathway | -0.34021814 | 0.00000000 |
| Atrazine degradation | 0.00000000 | -0.14804392 |
| Bacterial chemotaxis | 0.00000000 | -0.06444980 |
| Biosynthesis of siderophore group nonribosomal peptides | 0.00000000 | 0.36656274 |
| Drug metabolism cytochrome P450 | 0.07584680 | 0.45862556 |
| Inositol phosphate metabolism | 0.00000000 | 0.28993690 |
| Metabolism of xenobiotics by cytochrome P450 | 0.00000000 | 0.38812855 |
| Retinol metabolism | 0.00000000 | 0.62299766 |
| Tropane piperidine and pyridine alkaloid biosynthesis | 0.00000000 | -0.02421417 |
| Ubiquinone and other terpenoid quinone biosynthesis | 0.00000000 | 0.07618395 |
