# Supplementary material for: Transient effect of single or repeated acute deoxynivalenol and zearalenone dietary challenge on fecal microbiota composition in female finishing pigs
Source: Animal. 2020 Jun 18;14(11):2277–87. doi: 10.1017/S1751731120001299 (PMC7538342; doi:10.1017/S1751731120001299)
Supplement: Supplementary file 1 [file S1751731120001299sup.zip › S1751731120001299sup002.docx]

**Transient effect of single or repeated acute deoxynivalenol and zearalenone dietary challenge on fecal microbiota composition in female finishing pigs**

M. Le Sciellour, O. Zemb, A.-M. Serviento and D. Renaudeau

animal journal

Table S1 Composition of experiment diets^1^.

| Diet | Control | Mycotoxin-contaminated |
| --- | --- | --- |
| Ingredients, % as-fed |  |  |
| Corn | 75.00 | - |
| Corn, deoxynivalenol/zearalenone contaminated | - | 75.00 |
| Wheat bran | 1.54 | 1.64 |
| Soybean mean | 18.10 | 18.00 |
| Molasses | 2.00 | 2.00 |
| Lysine HCL | 0.21 | 0.21 |
| DL-Methionine | 0.08 | 0.08 |
| L-Threonine | 0.06 | 0.06 |
| L-Tryptophan | 0.01 | 0.01 |
| Dicalcium phosphate | 1.00 | 1.00 |
| Calcium carbonate | 1.00 | 1.00 |
| Salt | 0.50 | 0.50 |
| Vitamin-mineral premix^2^ | 0.50 | 0.50 |
|  |  |  |
| Calculated composition^3^ |  |  |
| Crude protein, % | 14.3 | 14.4 |
| Net energy, MJ/kg | 10.3 | 10.3 |
| SID Lysine, g/kg | 7.7 | 7.7 |
| SID Lysine/Net energy, g/MJ | 0.8 | 0.8 |
| SID Sulphuric amino acid, g/kg | 0.6 | 0.6 |
| SID Threonine, g/kg | 0.7 | 0.7 |
| SID Tryptophan, g/kg | 0.2 | 0.2 |
| Ca, g/kg | 7.8 | 6.5 |
| Digestible P, g/kg | 2.5 | 2.1 |
| Digestible P/Net energy, g/MJ | 0.3 | 0.3 |
| Ca/P digestible | 3.1 | 3.1 |
|  |  |  |
| Analyzed composition^4^ |  |  |
| Dry matter, % | 87.0 | 87.0 |
| Organic matter, % | 79.5 | 77.9 |
| Crude protein, % | 14.2 | 14.1 |
| Crude fat, % | 3.1 | 2.3 |
| Crude fiber, % | 1.5 | 1.7 |
| NDF, % | 6.3 | 7.5 |
| ADF, % | 1.8 | 2.0 |
| ADL, % | 0.3 | 0.2 |
| Starch, % | 50.6 | 50.7 |
| Growth energy, MJ/kg | 15.8 | 15.7 |
| Net energy, MJ/kg^5^ | 10.7 | 10.7 |
|  |  |  |
| Analyzed mycotoxin composition (mg/kg)^6^ |  |  |
| Deoxynivalenol | 0.14 | 3.02 |
| Nivalenol | 0.02 | 0.62 |
| Zearalenone | 0.10 | 0.76 |
| Fumonisin B1 | 0.45 | 0.06 |
| Fumonisin B2 | 0.10 | 0.01 |
| Aflatoxins B1, B2, G1 and G2 | <0.004 | <0.004 |

^1^Diet fed by the pigs in pellet form.

^2^Provided per kilogram of complete diet: vitamin A, 1 000 000 IU; vitamin D, 200 000 IU; vitamin E, 4 000 mg; vitamin B1, 400 mg; vitamin B2, 800 mg; calcium pantothenate, 2 170 mg; niacin, 3 000 mg; vitamin B12, 4 mg; vitamin B6, 200 mg; vitamin K3, 400 mg; folic acid, 200 mg; biotin, 40 mg; choline chloride, 100 000 mg; iron (sulfate), 11 200 mg; iron (carbonate), 4 800 mg; copper (sulfate), 2 000 mg; zinc (oxide), 20 000 mg; manganese (oxide), 8 000 mg; iodine (iodate), 40 mg; cobalt (carbonate), 20 mg; and selenium (selenite), 30 mg.

^3^As-fed basis. SID = standardized ileal digestible.

^4^As-fed basis. Values are calculated for the same dry matter content (87.0%).

^5^As-fed basis. Values are calculated based on equation set by Noblet et al. (1994, eq. 11) for calculating net energy (NE) in growing pigs.

^6^As-fed basis, calculation based on the dry matter content of 87%. Analyzed by a commercial laboratory (GIP Labocea, Ploufragan, FR).

Noblet J, Fortune H, Shi S and Dubois S 1994. Prediction of net energy value of feeds for growing pigs. Journal of Animal Science 72, 344–354.
