# Supplementary material for: Transient effect of single or repeated acute deoxynivalenol and zearalenone dietary challenge on fecal microbiota composition in female finishing pigs
Source: Animal. 2020 Jun 18;14(11):2277–87. doi: 10.1017/S1751731120001299 (PMC7538342; doi:10.1017/S1751731120001299)
Supplement: Supplementary file 1 [file S1751731120001299sup.zip › S1751731120001299sup003.docx]

**Transient effect of single or repeated acute deoxynivalenol and zearalenone dietary challenge on fecal microbiota composition in female finishing pigs**

M. Le Sciellour, O. Zemb, A.-M. Serviento and D. Renaudeau

*animal* journal

Table S2 Relative abundance (%) of families and genera in pigs’ fecal samples collected in the two different experimental groups^1^ at 140 days of age.

| Relative abundance (%) | CD | DD | p^2^ |
| --- | --- | --- | --- |
| Families |  |  |  |
| *Coriobacteriaceae* | 0.06 ± 0.04 | 0.03 ± 0.02 | <0.05 |
| *Lactobacillaceae* | 10.97 ± 10.39 | 17.02 ± 10.30 | <0.05 |
| *Spirochaetaceae* | 0.33 ± 0.28 | 0.43 ± 0.23 | <0.05 |
|  |  |  |  |
| Genera |  |  |  |
| *Collinsella* | 0.05 ± 0.03 | 0.03 ± 0.02 | <0.05 |
| *Lactobacillus* | 10.95 ± 10.35 | 16.96 ± 10.24 | <0.05 |

^1^The treatments groups are: the CD group fed a deoxynivalenol (DON)- and zearalenone (ZEN)-contaminated diet between 134 and 140 days, and the DD group fed a DON- and ZEN-contaminated diet between 113 and 119 days and between 134 and 140 days.

^2^The p-value (p) resulted from a Wilcoxon test. Only the families and genera that significantly differ between the treatments are presented in the table.
